# Two-Stage Surgical Management of Intramedullary Holocord Astrocytoma in an Adult: A Case Report and Literature Review

**DOI:** 10.3390/curroncol33010062

**Published:** 2026-01-21

**Authors:** Trong Huy Mai, Firat Taskaya, Sifian Al-Hamid, Julius Reiser, Vanessa Magdalena Swiatek, Ardeshir Ardeshiri, Ali Rashidi, Klaus-Peter Stein, Christian Mawrin, Belal Neyazi, I. Erol Sandalcioglu

**Affiliations:** 1 Department of Neurosurgery, Otto-von-Guericke University, 39120 Magdeburg, Saxony-Anhalt, Germany; 2 Institute of Neuropathology, Otto-von-Guericke University, 39120 Magdeburg, Saxony-Anhalt, Germany

**Keywords:** holocord astrocytoma, intramedullary spinal cord tumor, adult spinal glioma, two-stage resection, neuro-oncology

## Abstract

**Background/Objectives**: Holocord astrocytomas are exceptionally rare intramedullary tumors, especially in adults, and often present with extensive longitudinal growth. Because only a small number of cases have been described, management strategies remain insufficiently defined. This report presents an adult patient treated with a staged surgical approach and provides an updated review of the literature. **Methods**: A 31-year-old male presented with progressive paraparesis, sensory deficits, and sphincter dysfunction. MRI demonstrated an intramedullary tumor extending from T3 to the conus medullaris. The patient underwent a planned two-stage resection with intraoperative neurophysiological monitoring. Histopathological and DNA-methylation analyses were performed. Additionally, a systematic review of previously reported holocord astrocytoma cases was conducted. **Results**: The two-stage surgical strategy enabled extensive debulking across multiple spinal segments while preserving neurological function. The patient demonstrated marked postoperative improvement, including restoration of sphincter control, improved motor function, and better mobility. Histopathological analyses confirmed a high-grade astrocytoma with piloid features. The literature review identified 28 previously reported cases, including only 5 in adults. Reported neurological outcomes across adult cases are variable, reflecting the heterogeneity and rarity of this tumor entity. **Conclusions**: Holocord astrocytomas in adults are extremely rare and pose particular diagnostic and therapeutic challenges. This case demonstrates that a carefully planned, staged surgical approach can achieve meaningful neurological recovery, even in patients presenting with severe preoperative deficits. The report expands the limited body of evidence available for adult holocord astrocytomas and may support future management strategies.

## Simple Summary

Holocord astrocytomas are extremely rare spinal cord tumors, and only a few cases have been reported in adults. Because these tumors may extend along the entire spinal cord, diagnosis and treatment are often challenging. In this report, we describe a young adult who presented with severe neurological symptoms caused by such a tumor. We treated him using a planned two-stage surgical approach, allowing for safe removal of tumor tissue across multiple spinal levels. After surgery, the patient showed marked neurological improvement, regaining mobility and sphincter control. To contextualize this case, we also reviewed all published reports of holocord astrocytomas. Due to the small number of adult cases, clear treatment recommendations are limited. Our findings show that with careful planning, meaningful recovery can be achieved, and this report adds valuable clinical data to a very rare and understudied tumor entity.

## 1. Introduction

Spinal cord tumors are rare entities, with an incidence reported between 0.74 and 1.6 per 100,000 individuals annually. They represent approximately 15% of all tumors of the central nervous system [1,2,3,4]. Based on their anatomical localization, they are categorized as extradural, intradural–extramedullary, or intramedullary [5]. Among these, intramedullary tumors are the least frequent, accounting for about 10–30% of spinal tumors [1,6,7]. The majority of intramedullary tumors (around 80%) are of glial origin, while the remainder includes a heterogeneous group of other entities [4,7]. Ependymomas are the most common subtype (60–70%), followed by astrocytomas, which constitute about 30–35% [3,4,6,7,8].

Astrocytomas represent the second most common intramedullary tumor in adults and the leading one in children [9]. They typically arise in the cervical or thoracic cord and frequently involve multiple spinal segments [3,4]. The tumor shows a male predominance, with incidence peaks during childhood and between the ages of 30 and 50 [3]. Histopathological grading follows the WHO classification applied to cerebral astrocytomas [10]. While most intramedullary astrocytomas are low-grade (WHO-grades 1–2), approximately 25% present as high-grade tumors (WHO-grades 3–4), which are associated with a significantly poorer prognosis [11]. Pilocytic astrocytomas are more frequently observed in pediatric patients, whereas malignant progression occurs more often in adults [10].

The clinical course is often insidious, and symptoms are nonspecific, which frequently delays diagnosis. Early magnetic resonance imaging (MRI) of the spinal axis plays a critical role in establishing the diagnosis and initiating treatment. The most common presenting features include back pain, sensory disturbances, and motor weakness, while bladder or bowel dysfunction tends to occur later in the disease course. The extent and level of spinal cord involvement largely determine the clinical manifestations [4]. High-resolution sagittal and axial MRI sequences of the whole spinal cord represent the diagnostic standard for intramedullary lesions [3,4,7].

A distinct and particularly challenging variant within the spectrum of intramedullary astrocytomas is the holocord astrocytoma, which extends across the entire length of the spinal cord. These tumors pose considerable therapeutic difficulties. Various surgical approaches have been reported, ranging from extended midline myelotomy with biopsy and laminoplasty [9] to gross total resection [12], both presenting favorable outcomes in pediatric patients. Ebner et al. recommend that, in both adults and children, an attempt should be made to achieve total resection, either in a single session or a staged procedure, with the support of intraoperative neurophysiological monitoring [13], which, however, may be of limited utility in severe cases. Adjuvant radiotherapy or chemotherapy is generally reserved for recurrent disease [13,14]. Due to their exceptional rarity, only a handful of holocord astrocytomas have been reported in the literature, with merely five cases documented in adults.

We report the case of a 31-year-old male with a rare holocord intramedullary astrocytoma extending from T3 to the conus medullaris, presenting with progressive neurological decline nearly a decade after initial shunt treatment. This case highlights the challenges of surgical management in adults and adds to the limited literature on this exceptional tumor entity. In addition to reporting this case, we conducted a systematic review of the literature to place our findings in the context of all previously documented holocord astrocytomas. A structured search of PubMed, EMBASE, and CENTRAL identified reports published up to June 2025.

## 2. Materials and Methods

### 2.1. Case Report

A 31-year-old male first presented in 2015 at a medical center abroad with progressive gait instability, muscle weakness, and urinary incontinence. MRI revealed intramedullary thoracic spinal cord masses associated with a large syrinx. Surgical resection was deferred at that time, and the patient underwent symptomatic treatment with placement of a syringo-subarachnoid shunt, resulting in marked regression of symptoms and clinical improvement.

#### 2.1.1. Clinical Presentation

From 2023 onward, the patient experienced recurrent and progressively worsening symptoms. At the time of admission to the Department of Neurosurgery, University Hospital Magdeburg, in February 2025, he had been unable to ambulate for four months, even with assistive devices. Neurological examination revealed paraparesis with motor strength graded 2/5 in the right and 3/5 in the left lower extremity. Hypesthesia was present from the T12 dermatome downward. Hyperreflexia and clonus were noted in the right lower limb, and Babinski signs were bilaterally positive. Additionally, the patient reported recurrent bladder and bowel dysfunction. Neurological function was further assessed using the Modified McCormick Scale (MMS) [15,16], corresponding to grade IV, and the Klekamp–Samii Score (KSS) [17,18], corresponding to 8.

#### 2.1.2. Imaging Findings

**Figure 1:**
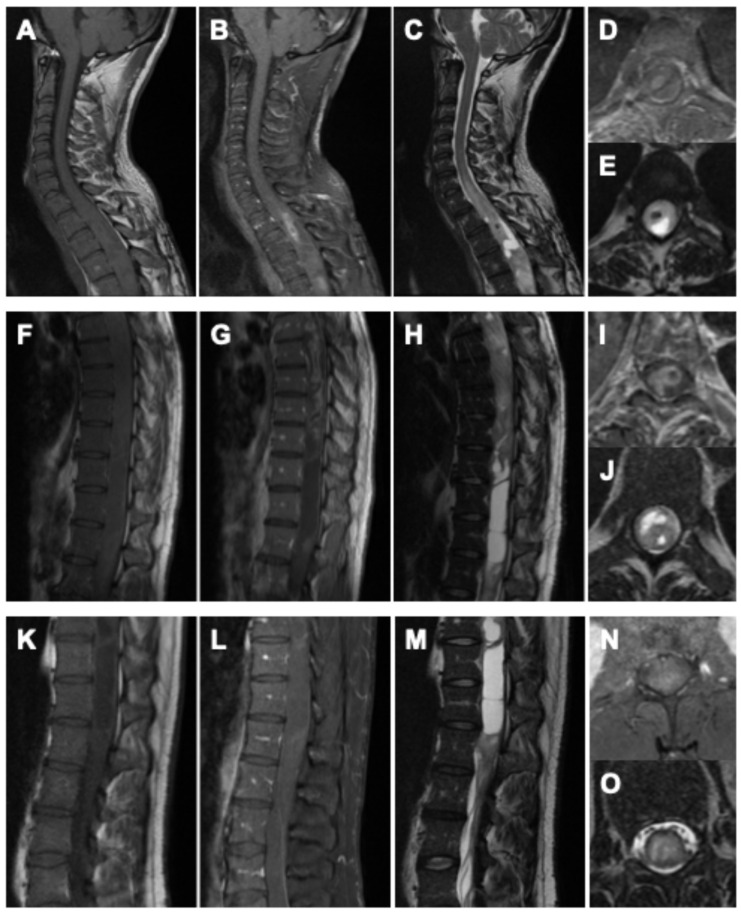
Preoperative imaging of the holocord astrocytoma. Cervical spine: (**A**) sagittal native T1-weighted MRI, (**B**) sagittal gadolinium-enhanced T1-weighted MRI, (**C**) sagittal native T2-weighted MRI, (**D**) axial gadolinium-enhanced T1-weighted MRI, and (**E**) axial native T2-weighted MRI. Thoracic spine: (**F**) sagittal native T1-weighted MRI, (**G**) sagittal gadolinium-enhanced T1-weighted MRI, (**H**) sagittal native T2-weighted MRI, (**I**) axial gadolinium-enhanced T1-weighted MRI, and (**J**) axial native T2-weighted MRI. Lumbar spine: (**K**) sagittal native T1-weighted MRI, (**L**) sagittal gadolinium-enhanced T1-weighted MRI, (**M**) sagittal native T2-weighted MRI, (**N**) axial gadolinium-enhanced T1-weighted MRI, and (**O**) axial native T2-weighted MRI.

Whole-spine MRI demonstrated a heterogeneous, contrast-enhancing intramedullary tumor extending from T3 to the conus medullaris, associated with marked cord expansion and a multi-chambered syrinx. On T1-weighted sequences, the lesion appeared predominantly hyperintense with diffuse and partly nodular enhancement. Corresponding T2-weighted images revealed extensive hyperintense signal alterations with indistinct tumor–cord boundaries in several regions, as well as syrinx cavities spanning the thoracic and lumbar cord. Multifocal areas of hemorrhage were noted in the thoracic segments. In addition, a progressive T2-hyperintense signal without contrast enhancement was observed from C6 caudally, reflecting further myelon involvement at the cervical level. At the lumbar spine, an expansive intramedullary lesion centered at L1/2 with diffuse contrast uptake, and associated syrinx formation was identified, accompanied by additional hyperintense epidural formations, considered most likely to represent CSF loculations secondary to disturbed liquor circulation (Figure 1).

As part of the preoperative staging, cranial MRI and CSF cytology were performed prior to the first operation and showed no evidence of intracranial tumor manifestation or malignant cells.

#### 2.1.3. Surgical Strategy and Intraoperative Management

The case was presented at the institutional neuro-oncological tumor board, where a spinal malignant astrocytoma was suspected. Given the patient’s neurological deterioration, partial tumor resection with histological confirmation was planned as the first stage of the surgical procedure. A planned two-stage surgical approach was undertaken, and both procedures were performed under continuous intraoperative electrophysiological monitoring. The first stage focused on histopathological confirmation and the second on tumor control and functional recovery. In this case, however, intraoperative monitoring was of limited usefulness owing to the severity of the neurological deficits.

##### First-Stage Procedure

**Figure 2:**
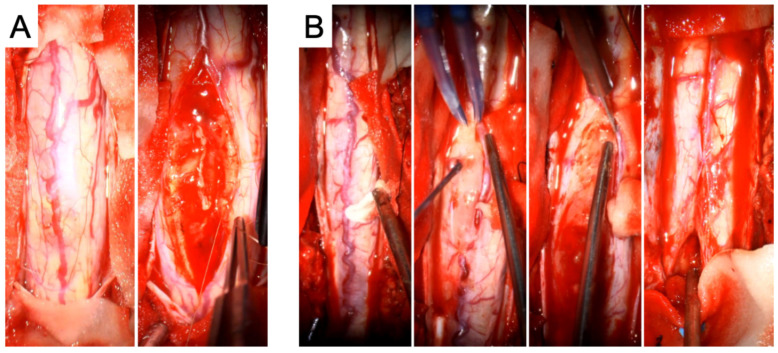
Intraoperative images from the first tumor resection (**A**) and the second tumor resection (**B**). In (**A**), the initial view of the spinal cord is shown, followed by the macroscopic morphology of the tumor. In (**B**), the surgical strategy is illustrated in greater detail: first, the exposure of the spinal cord is shown, followed by a macroscopic view of the tumor and preparation of the tumor–spinal cord interface, which is also depicted in the subsequent imaging. Finally, closure of the spinal cord by arachnoid suturing after complete tumor resection is demonstrated.

During the first operation, a laminectomy from T3 to T6 with midline myelotomy and tumor resection was carried out, accompanied by partial removal of the previously placed syringo-subarachnoid shunt catheter. Intraoperatively, distinct tumor–spinal cord margins were identifiable in certain areas, whereas in other regions, the transition was indistinct (Figure 2).

**Figure 3:**
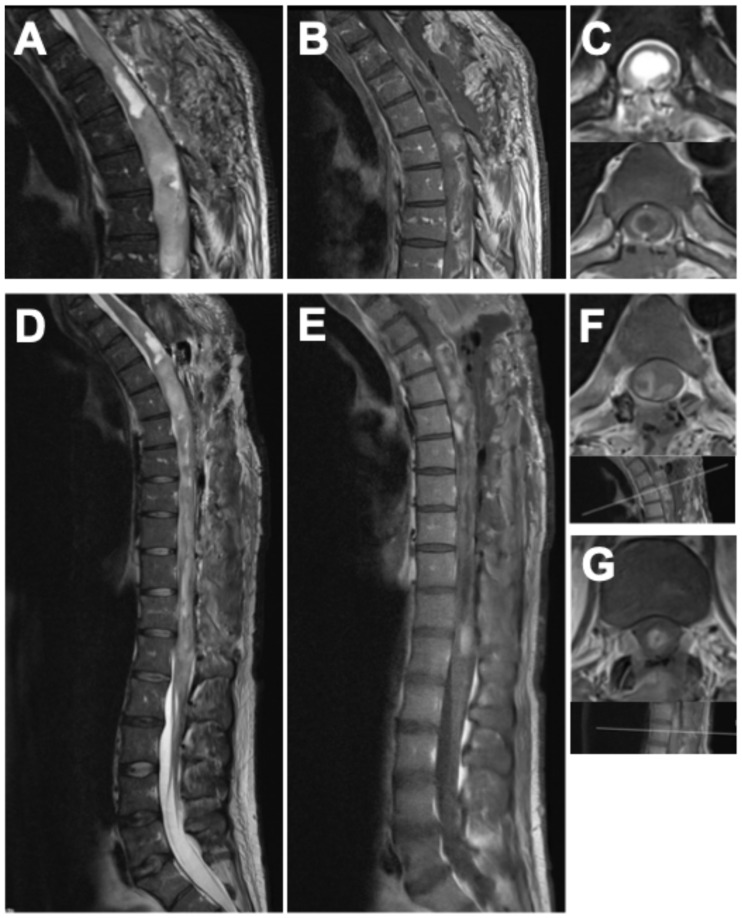
Postoperative MRI of the spine after the first surgery (**A**–**C**), showing the thoracic spine in (**A**) sagittal T2-weighted, (**B**) sagittal gadolinium-enhanced T1-weighted, and (**C**) axial T2- and gadolinium-enhanced T1-weighted sequences. Postoperative findings after the second surgery are displayed in (**D**–**G**): (**D**) sagittal T2-weighted, (**E**) sagittal gadolinium-enhanced T1-weighted, (**F**) axial gadolinium-enhanced T1-weighted MRI of the thoracic spine with indication of the imaging plane, and (**G**) axial gadolinium-enhanced T1-weighted MRI at the thoracolumbar junction with corresponding imaging plane. Following the second procedure, postoperative MRI confirmed only minimal residual tumor at the levels of T5–T7 and T12/L1, showing marked regression compared with preoperative imaging, accompanied by substantial decompression and regression of the syrinx.

Postoperatively, the patient demonstrated immediate neurological improvement, with motor strength of 3/5 in both lower extremities and recovery of sphincter function. Spasticity and gait disturbance persisted. The postoperative neurological status corresponded to MMS grade IV and KSS 10. MRI following the first surgery revealed diffuse intra-axial contrast enhancement between T3 and T6, residual tumor tissue, and syrinx cavities extending to the conus medullaris (Figure 3A–C).

Intraoperatively and on conventional histopathological assessment, the tumor exhibited features suggestive of a low-grade astrocytic lesion, including low mitotic activity, absence of necrosis, and a low proliferation index. However, despite these seemingly indolent characteristics, DNA-methylation profiling classified the tumor as a high-grade astrocytoma with piloid features (CNS WHO-grade 3). This discrepancy between histological appearance and molecular classification indicated a biologically more aggressive entity. The case was re-evaluated at the institutional neuro-oncological tumor board, which recommended further debulking of the residual tumor.

##### Second-Stage Procedure

Twelve weeks later, a second procedure was performed, consisting of extended tumor resection from T7 to L1 and complete removal of the remaining shunt catheter (Figure 2). Tumor exposure was achieved via multilevel laminotomies (T6–T12); the laminae were preserved and reinserted at the end of the procedure to maintain the integrity of the posterior spinal elements. The operation proceeded without complications. Neurological examination after the second stage demonstrated substantial functional improvement compared with the preoperative baseline. Muscle strength had increased to 4–4+/5 in both lower extremities, sphincter function was fully restored, and the patient was able to stand with external support. The MMS improved to grade III and the KSS to 16, accompanied by a clear improvement in back and spinal pain control, facilitating mobilization. Postoperative MRI confirmed only minimal residual tumor at the levels of T5–T7 and T12/L1, showing marked regression compared with the preoperative imaging (Figure 3D–G).

##### Follow-Up

For follow-up, the multidisciplinary tumor board recommended a watch-and-wait strategy rather than adjuvant radiotherapy. This decision was based on the predominantly low-grade histological features, the absence of further surgical resection options, and the patient’s severely impaired neurological status prior to surgery, with ongoing postoperative recovery despite clinical improvement. The aim was to allow for further neurological recovery and avoid premature overtreatment while maintaining close surveillance. The patient was referred for postoperative rehabilitation and scheduled for clinical and radiological re-evaluation at three months, including MRI of the spine and CT imaging to assess tumor status and spinal alignment. Unfortunately, the patient was lost to follow-up before the planned three-month evaluation could be performed.

#### 2.1.4. Histopathological and Molecular Analysis

Intraoperatively, the tumor appeared as a beige-reddish colored, very soft mass compatible with a glioma. Histopathological examinations demonstrated pleomorphic tissue with moderate cell density, with bipolar cell processes and individual Rosenthal fibers. In some areas, vascular proliferations were present. There was no evidence of necrosis or a mitotic increase.

Formalin-fixed, paraffin-embedded tumor tissue was evaluated by hematoxylin and eosin staining and immunohistochemistry for glial fibrillary acidic protein (GFAP), microtubule-associated protein 2 (MAP2), neurofilament (NF), alpha-thalassemia/mental retardation syndrome X-linked protein (ATRX), oligodendrocyte transcription factor 2 (OLIG2), p53, epithelial membrane antigen (EMA), mutant IDH1 (R132H), and mindbomb E3 ubiquitin protein ligase 1 (MIB1). Immunohistochemical analysis revealed diffuse positivity for GFAP. MAP2 was expressed in a subset of tumor cells, whereas NF staining highlighted only pre-existing peripheral fiber connections. ATRX expression was largely preserved with nuclear staining, and the tumor cells demonstrated nuclear positivity for OLIG2. Immunostaining using an antibody against mutant IDH1 (R132H) showed no immunopositivity. EMA was not expressed, and p53 showed no relevant nuclear accumulation, with only very rare immunopositive nuclei. The proliferation index, assessed by MIB1, indicated approximately 3–5% immunopositive nuclei. Based on the initial microscopic assessment, a low-grade astrocytic tumor was suspected.

Regarding molecular genetic testing, Sanger sequencing of exon 15 of the B-raf proto-oncogene, serine/threonine kinase (BRAF) gene was performed from tumor tissue, and no mutation at BRAF codon 600 was detected. With respect to additional clinically relevant alterations, H3K27M status was not assessed in the available diagnostic work-up. Targeted sequencing for other MAPK pathway alterations beyond BRAF codon 600 was not performed, and no additional targeted molecular panel results are available.

**Figure 4:**
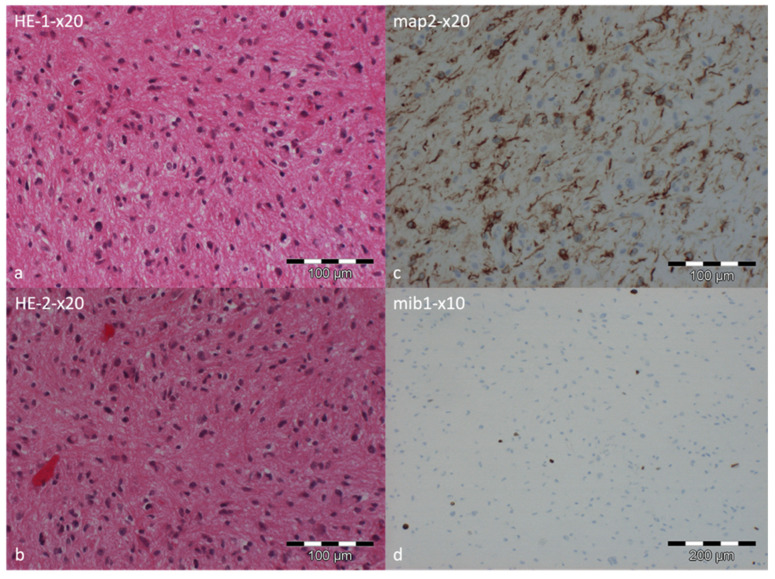
Histopathological examination of the tumor specimen. Hematoxylin and eosin staining at low magnification showing slightly to moderately cellular astroglial tumor tissue with minimal pleomorphism. Tumor cells exhibit partly bipolar processes, and occasional Rosenthal fibers are noted. Focal areas of small vascular proliferations are present (**a**,**b**). The neuronal differentiation marker MAP2 was expressed in a subset of tumor tissue, highlighting axon-bearing tumor cells (**c**). The proliferation marker MIB1 indicated approximately 3% immunopositive nuclei (**d**).

Genome-wide DNA methylation profiling was performed using the Illumina EPIC 935K v2.0 BeadChip (San Diego, CA, USA) on DNA extracted from formalin-fixed, paraffin-embedded tumor tissue. The methylation profile was compared with reference methylation profiles from more than 7495 cases across 184 tumor classes using the Brain Tumor Classifier, version v12.8 (https://app.epignostix.com, accessed on 4 August 2025). The DNA methylation profile was consistent with the methylation class “high-grade astrocytoma with piloid features”, with a classifier score of 0.95 (cut-off: 0.9). As part of the EPIC analysis, copy-number variation profiling suggested the presence of a CDKN2A/B deletion. In addition, MGMT promoter methylation was assessed and demonstrated a methylated status with a score of 0.92144 (cut-off: 0.3582). Therefore, the DNA methylation profiling classified the lesion as compatible with a high-grade astrocytoma with piloid features (CNS WHO-grade 3), with a classifier score of 0.95 (cut-off: 0.9), indicating a high-grade entity despite its low proliferative activity (Figure 4).

### 2.2. Review of the Literature

#### 2.2.1. Search Strategy

To complement our findings, we performed a structured literature search in PubMed, EMBASE, and CENTRAL for reports published up to June 2025, focusing on holocord astrocytoma. The search strategy combined the keyword “holocord” with “astrocytoma” or “intramedullary glioma”. To minimize the risk of missing relevant cases, reference lists of all eligible publications were manually screened for additional reports.

#### 2.2.2. Eligibility Criteria

Reports were eligible for inclusion if they provided histopathological confirmation of holocord astrocytoma and included relevant clinical information regarding presentation, treatment, or outcome. Publications were required to be peer-reviewed journal articles or book chapters. To avoid language bias, non-English publications were also considered. Reports describing holocord tumors of other histological subtypes or lacking histopathological confirmation were excluded.

#### 2.2.3. Data Extraction and Synthesis

All cases meeting the predefined inclusion criteria were incorporated into the qualitative synthesis. Extracted data included patient demographics, tumor extent, histopathological diagnosis, surgical strategy, use of adjuvant therapies, and reported neurological outcomes. Given the rarity of the entity and the heterogeneity of available reports, data were summarized descriptively without formal meta-analysis.

## 3. Results

### 3.1. Demographic and Tumor Characteristics

To date, 28 cases of holocord astrocytoma have been reported in the literature [9,12,13,14,19,20,21,22,23,24,25,26,27,28,29,30,31,32,33,34,35]. Of these, 17 (60.7%) were male, and 9 (31%) were female; sex was not mentioned in 2 cases (6.9%). The mean age at diagnosis was 11.2 years (SD = 9.2; age range: 3 weeks–29 years). In total, 93% of the cases were low-grade astrocytoma (WHO-grade 1 and 2), while in two cases (7%), tumor grading was not further specified.

Across the literature, only five cases in individuals older than 18 years have been described. Here, the mean age was 26.4 (SD = 2.8; age range: 23–29 years). Accordingly, this case adds to the literature as the 29th reported holocord astrocytoma and the 6th identified in the adult population. A summary of reported cases is presented in Table 1.

### 3.2. Preoperative Neurological Status

Preoperative neurological status according to the MMS was reported in 20 cases. Five patients (17.9%) presented with MMS grade II, three (10.7%) with grade III, eleven (39.2%) with grade IV, and one patient (3.6%) with grade V. Preoperative KSS values were available in 17 cases, with a mean score of 15 (range 3–25).

Within the adult subgroup, only one patient presented with a favorable neurological status (MMS grade II), whereas the remaining four adult patients were classified as MMS grade IV. Preoperative KSS values in adults ranged from 9 to 23, with a mean of 14, indicating advanced neurological impairment at diagnosis.

### 3.3. Surgical Management and Adjuvant Treatment

Information on surgical treatment was unavailable in three cases. Among the remaining patients, 3 underwent biopsy only, 14 underwent subtotal resection, and 8 underwent gross total resection. Data on shunt placement were missing in seven cases. Of the documented cases, 15 patients (71.4%) did not require cerebrospinal fluid diversion, while 6 patients (28.6%) underwent shunt placement (five ventriculoperitoneal shunts and one syringo-subarachnoid shunt).

Radiotherapy data were available in 22 cases, of which 6 patients (27.3%) received radiotherapy (3 preoperatively and 3 postoperatively), while 16 patients (72.7%) did not. Chemotherapy information was reported in 20 cases; 5 patients (25%) received chemotherapy (2 treated with vincristine–carboplatin, 1 with temozolomide, and 2 with unspecified regimens), whereas 15 patients (75%) did not.

In the adult subgroup, only one patient underwent gross total resection, while all others received subtotal resection. Two adult patients required CSF diversion (one ventriculoperitoneal shunt and one syringo-subarachnoid shunt). Two adult patients received radiotherapy, whereas none underwent chemotherapy.

### 3.4. Postoperative Neurological Outcomes

Postoperative neurological status according to the MMS was reported in 21 cases. Six patients (21.4%) achieved MMS grade I, nine (32.1%) grade II, one (3.6%) grade III, four (14.3%) grade IV, and one (3.6%) grade V. Postoperative KSS values were available in 15 cases, with a mean score of 19 (range: 2–24). Information on postoperative neurological dynamics was unavailable in three cases.

Overall, information on postoperative outcomes compared to preoperative neurological status was reported in 25 cases. Fourteen patients (66.7%) demonstrated neurological improvement, nine patients (42.9%) remained neurologically stable, one patient (4.8%) deteriorated, and one patient (4.8%) died.

In the adult subgroup, postoperative MMS was not reported in one case. Of the remaining patients, two achieved MMS grade I, one grade II, and one grade IV. Postoperative KSS values were unavailable in one case; among the remaining adults, the mean postoperative KSS was 20 (range 14–24). Regarding neurological course, two adult patients improved, one remained stable, and one deteriorated.

## 4. Discussion

Holocord astrocytomas represent an exceptionally rare variant within the spectrum of intramedullary spinal cord tumors. Our review identified 28 previously published cases, of which only 5 involved adult patients. With the present case, we add the 29th overall and the 6th case in an adult, thereby contributing to a particularly underrepresented subgroup.

Consistent with prior reports, our patient presented with advanced neurological deficits, including paraparesis and sphincter dysfunction, reflected by a preoperative MMS grade IV and KSS of 8. This presentation closely mirrors the typical clinical profile reported in the literature, where most patients with holocord astrocytomas present with severe neurological compromise, as 77% had MMS grade III–IV at diagnosis, while only 18% had MMS grade II. Within the adult subgroup, four of five previously reported patients presented with MMS grade IV [20,21,22,26], underscoring the aggressive clinical course and diagnostic delay often seen in adults.

Postoperative outcomes across all reported cases indicate meaningful functional improvement in the majority, with 67% showing neurological recovery and an additional 43% achieving stabilization. In adults, the course is less favorable: while two patients improved [22,26], one remained stable [27], and one worsened [20], reflecting the difficulty of achieving significant recovery once advanced deficits have been established. Our patient, however, demonstrated considerable postoperative improvement, regaining sphincter function and achieving functional gains to MMS grade III and KSS 16. This compares favorably with outcomes reported in prior adult cases and highlights the potential benefit of carefully staged surgical intervention.

The optimal surgical management of holocord astrocytomas remains a matter of debate. In the literature, gross total resection has been reported in eight cases overall but was achieved in only one adult [22]. Most adult patients underwent subtotal resection [20,21,26,27], often due to indistinct tumor–cord boundaries and the risk of severe morbidity. In our case, we opted for a two-stage resection strategy, enabling safe debulking across an extended tumor length (T3–L1). This approach allowed for partial resection during the first surgery with preservation of function, followed by a second-stage resection that further reduced tumor mass while minimizing neurological risk. This strategy directly addresses the challenge of extensive tumor length and indistinct tumor–cord interfaces frequently reported in adult cases. Postoperative MRI demonstrated only minimal residual tumor, with marked regression of the syrinx. Compared with previous adult reports, our surgical approach is notable for its staged nature, which likely contributed to the favorable functional outcome.

The role of adjuvant radiotherapy or chemotherapy remains controversial. In our review, 27% of patients received radiotherapy [20,24,25,26,28], either pre- or postoperatively, and 25% received chemotherapy [13,19,23,25,28], most often vincristine and carboplatin in pediatric patients. Among adult cases, two patients underwent radiotherapy [22,26], but none received chemotherapy. In our patient, adjuvant treatment was deferred in favor of a watch-and-wait strategy, in line with current recommendations that reserve radiotherapy for progressive or recurrent disease, particularly in low-grade tumors. Notably, the discrepancy between low-grade histological features and high-grade molecular classification in our case underscores the growing importance of molecular profiling in intramedullary tumors and may have implications for surgical decision-making and follow-up strategies.

The rarity of holocord astrocytomas in adults makes it difficult to derive evidence-based treatment guidelines. Compared to pediatric cases, adults are more often diagnosed at advanced functional impairment, as also reflected in our patient; rarely undergo gross total resection; and show less pronounced neurological recovery. Our case demonstrates that with careful planning, staged surgery, and intraoperative monitoring, meaningful functional improvement is achievable even in this challenging subgroup.

Our case reflects the limitations inherent to rare tumor entities: the absence of standardized treatment algorithms, reliance on case-based experience, and the challenge of balancing surgical radicality with functional preservation. Further multicenter collaboration and pooled analyses will be essential to refine management strategies for adult patients with holocord astrocytomas.

## 5. Conclusions

Holocord astrocytomas are extremely rare, particularly in adults, and are frequently associated with severe neurological deficits at diagnosis. Our literature review demonstrates that adults typically undergo subtotal resection and achieve limited functional recovery. In contrast, our case illustrates that a staged surgical strategy can result in meaningful neurological improvement and possible oncological control. This highlights the importance of individualized surgical planning and contributes valuable evidence to the scarce literature on adult holocord astrocytomas.

## Figures and Tables

**Table 1:** Overview of reported cases of holocord astrocytoma, summarizing patient demographics, tumor extent, treatment strategies, and pre- and postoperative neurological outcomes assessed by the Modified McCormick Scale (MMS) and the Klekamp–Samii Score (KSS). Adult cases (≥18 years) are highlighted.

Study	Age	Sex	Diagnosis	Extent of Tumor	Resection	Shunt	RTx	CTx	PostOP Outcome	MMS preOP	MMS postOP	KSS preOP	KSS postOP
Epstein and Epstein [24]	4	M	Low-grade astrocytoma	CMJ– Conus	STR	No	Yes, preoperative	No	Neurological improvement	IV	I	19	25
	5	M	Low-grade astrocytoma	CMJ– Conus	STR	No	Yes, preoperative	No	Neurological improvement	IV	II	11	24
	15	M	Low-grade astrocytoma	CMJ– Conus	STR	No	No	No	Neurological improvement	IV	IV	11	19
Tedeschi et al. [34]	12	F	Astrocytoma WHO-grade 2	C1–L2	STR	N/A	N/A	No	Neurological improvement	V	V	3	4
Nikaido et al. [31]	2	M	Astrocytoma	C4– Conus	GTR	N/A	N/A	N/A	Death				
Benzel et al. [22]	**23**	**M**	**Low-grade** **astrocytoma**	**C1**–**L2**	**GTR**	**N/A**	**Yes,** **postoperative**	**No**	**Neurological improvement**	**IV**	**II**	**14**	**23**
	30 months	M	Low-grade astrocytoma	C1– L3	GTR	N/A	No	No	Neurological improvement		II		22
Vles et al. [35]	11	F	Pilocytic astrocytoma WHO-grade 1	CMJ– S2	Biopsy	No	N/A	N/A	Neurological stabilization	IV	IV		
Irikura et al. [26]	**27**	**M**	**Astrocytoma**	**CMJ**–**Conus**	**STR**	**SSS**	**Yes**	**No**	**Neurological improvement**	**IV**	**I**	**9**	**23**
Shafrir and Kaufman [33]	4 months	M	Low-grade astrocytoma	C3– T12	N/A	N/A	N/A	N/A	Neurological stabilization				
Minehan et al. [30]	N/A	N/A	Pilocytic astrocytoma WHO-grade 1	Holocord	N/A	N/A	N/A	N/A	N/A				
Lau et al. [29]	4 months	M	Low-grade astrocytoma	Holocord	N/A	N/A	N/A	N/A	N/A				
Chacko and Chandy [12]	14	M	Astrocytoma WHO-grade 2	CMJ– L1	GTR	No	No	No	Neurological improvement	III	II	16	21
Sandalcioglu et al. [9]	4 months	M	Pilocytic astrocytoma WHO-grade 1	CMJ– Conus	STR	VPS	No	No	Neurological stabilization, Progression on MRI		II	25	20
Komotar et al. [28]	6	M	Pilomyxoid astrocytoma WHO-grade 2	C2– T11	STR	No	Yes	Yes	Neurological stabilization, Residue on MRI	III	I	22	24
	3 weeks	M	Pilomyxoid astrocytoma WHO-grade 2	CMJ– T12	Biopsy	VPS	No	N/A	Neurological stabilization, Progression on MRI				
Tobias et al. [14]	11	F	Pilocytic astrocytoma WHO-grade 1	C6– L1	GTR	No	No	N/A	Neurological improvement	II	III		
	13	M	Pilocytic astrocytoma WHO-grade 1	C4– L1	GTR	No	No	N/A	Neurological improvement	II	II		
Schittenheim et al. [32]	13	F	Pilocytic astrocytoma WHO-grade 1	CMJ– Conus	STR	VPS	No	No	Neurological stabilization, Progression on MRI	IV	II		
Ebner et al. [13]	10	M	Pilocytic astrocytoma WHO-grade 1	Medulla–Conus	GTR	No	No	No	Neurological improvement above T1	II	IV	18	2
	13	F	Pilocytic astrocytoma WHO-grade 1	CMJ– Conus	STR	No	No	No	Neurological stabilization, Progression on MRI	IV	II	12	19
	11	F	Pilocytic astrocytoma WHO-grade 1	Medulla– L2	STR	VPS	No	Vincristine + carboplatin	Neurological stabilization, Progression on MRI	II	II	19	
Goyal et al. [25]	14	F	Astrocytoma WHO-grade 2	CMJ– L1	Biopsy	No	Yes	Temozolomide	Neurological improvement, Residue on MRI	IV	I	7	
Bansal et al. [20]	**29**	**M**	**Pilocytic** **astrocytoma WHO-grade 1**	**CMJ**–**T10**	**STR**	**VPS**	**No**	**No**	**Neurological worsening**	**IV**	**IV**	**16**	**14**
Baran et al. [21]	**29**	**F**	**Pilocytic** **astrocytoma WHO-grade 1**	**C4**–**Conus**	**STR**	**No**	**No**	**No**	**N/A**	**IV**		**9**	
Arshad et al. [19]	14	M	Pilocytic astrocytoma WHO-grade 1	C1–Conus	STR	No	No	Yes	Neurological improvement	III	I	19	24
Salmi et al. [27]	**24**	**F**	**Pilocytic** **astrocytoma WHO-grade 1**	**Holocord**	**STR**	**No**	**No**	**No**	**Neurological stabilization, Residue on MRI**	**II**	**I**	**23**	**24**
Dolen et al. [23]	11 months	N/A	Pilocytic astrocytoma WHO-grade 1	CMJ–Conus	GTR	No	No	Vincristine + carboplatin	Neurological improvement				

Adult cases are highlighted in bold text. The underlined MMS and KS values were explicitly stated by the original authors in their reports. M: male; F: female; WHO: World Health Organization; N/A: data not available or not reported; CMJ: cervicomedullary junction; GTR: gross tumor resection; STR: subtotal tumor resection; RTx: radiotherapy; CTx: chemotherapy; SSS: syringo-subarachnoid shunt; VPS: ventriculo-peritoneal shunt.

## Data Availability

The data presented in this study are available in this article.

## Abbreviations

The following abbreviations are used in this manuscript:

ATRX	Alpha-thalassemia/mental retardation syndrome X-linked protein
BRAF	B-raf proto-oncogene, serine/threonine kinase
CSF	Cerebrospinal fluid
CTx	Chemotherapy
EMA	Epithelial membrane antigen
FLAIR	Fluid-attenuated inversion recovery
GFAP	Glial fibrillary acidic protein
KSS	Klekamp–Samii Score
MAP2	Microtubule-associated protein 2
MIB1	Mindbomb E3 ubiquitin protein ligase 1
MMS	Modified McCormick Scale
MRI	Magnetic resonance imaging
NF	Neurofilament
OLIG2	Oligodendrocyte transcription factor 2
R132H	Mutant IDH1
RTx	Radiotherapy
SD	Standard deviation
SSS	Syringo-subarachnoid shunt
VPS	Ventriculo-peritoneal shunt
WHO	World Health Organization

## References

[1] Duong L.M., McCarthy B.J., McLendon R.E., Dolecek T.A., Kruchko C., Douglas L.L., Ajani U.A. (2012). Descriptive Epidemiology of Malignant and Nonmalignant Primary Spinal Cord, Spinal Meninges, and Cauda Equina Tumors, United States, 2004–2007. Cancer.

[2] Schellinger K.A., Propp J.M., Villano J.L., McCarthy B.J. (2008). Descriptive Epidemiology of Primary Spinal Cord Tumors. J. Neuro-Oncol..

[3] Ottenhausen M., Ntoulias G., Bodhinayake I., Ruppert F.-H., Schreiber S., Förschler A., Boockvar J.A., Jödicke A. (2019). Intradural Spinal Tumors in Adults-Update on Management and Outcome. Neurosurg. Rev..

[4] Samartzis D., Gillis C.C., Shih P., O’Toole J.E., Fessler R.G. (2015). Intramedullary Spinal Cord Tumors: Part I-Epidemiology, Pathophysiology, and Diagnosis. Global Spine J..

[5] Mechtler L.L., Nandigam K. (2013). Spinal Cord Tumors: New Views and Future Directions. Neurol. Clin..

[6] Neyazi B., Haghikia A., Mawrin C., Hattingen E., Vordermark D., Sandalcioglu I.E. (2024). Spinal Intramedullary Tumors. Dtsch. Arztebl. Int..

[7] Van Goethem J.W.M., van den Hauwe L., Ozsarlak O., De Schepper A.M.A., Parizel P.M. (2004). Spinal Tumors. Eur. J. Radiol..

[8] Grimm S., Chamberlain M.C. (2009). Adult Primary Spinal Cord Tumors. Expert Rev. Neurother..

[9] Sandalcioglu I.E., Gasser T., Wiedemayer H., Horsch S., Stolke D. (2002). Favourable Outcome after Biopsy and Decompression of a Holocord Intramedullary Spinal Cord Astrocytoma in a Newborn. Eur. J. Paediatr. Neurol..

[10] Louis D.N., Perry A., Wesseling P., Brat D.J., Cree I.A., Figarella-Branger D., Hawkins C., Ng H.K., Pfister S.M., Reifenberger G. (2021). The 2021 WHO Classification of Tumors of the Central Nervous System: A Summary. Neuro Oncol..

[11] Ardeshiri A., Chen B., Hütter B.-O., Oezkan N., Wanke I., Sure U., Sandalcioglu I.E. (2013). Intramedullary Spinal Cord Astrocytomas: The Influence of Localization and Tumor Extension on Resectability and Functional Outcome. Acta Neurochir..

[12] Chacko A.G., Chandy M.J. (2000). Favorable Outcome after Radical Excision of a “Holocord” Astrocytoma. Clin. Neurol. Neurosurg..

[13] Ebner F.H., Schittenhelm J., Roser F., Scheel-Walter H., Tatagiba M., Schuhmann M.U. (2012). Management of Holocord Pilocytic Astrocytomas in Children and Adolescents: An Update. Pediatr. Neurosurg..

[14] Tobias M.E., McGirt M.J., Chaichana K.L., Goldstein I.M., Kothbauer K.F., Epstein F., Jallo G.I. (2008). Surgical Management of Long Intramedullary Spinal Cord Tumors. Childs Nerv. Syst..

[15] McCormick P.C., Stein B.M. (1990). Intramedullary Tumors in Adults. Neurosurg. Clin. N. Am..

[16] McCormick P.C., Torres R., Post K.D., Stein B.M. (1990). Intramedullary Ependymoma of the Spinal Cord. J. Neurosurg..

[17] Klekamp J., Samii M. (1993). Introduction of a Score System for the Clinical Evaluation of Patients with Spinal Processes. Acta Neurochir..

[18] Samii M., Klekamp J. (1994). Surgical Results of 100 Intramedullary Tumors in Relation to Accompanying Syringomyelia. Neurosurgery.

[19] Arshad A., Hanif S., Yusuf I., Hussain K. (2021). First Local Case Report of a Holocord Pilocytic Astrocytoma—An Uncommon Entity with Management Challenges. Pak. J. Neurol. Surg..

[20] Bansal S., Borkar S.A., Mahapatra A.K. (2017). Hydrocephalus Associated with Spinal Intramedullary Pilocytic Astrocytoma. Asian J. Neurosurg..

[21] Baran O., Kasimcan O., Sav A., Oruckaptan H. (2019). Holocord Pilocytic Astrocytoma in an Adult: A Rare Case Report and Review of the Literature. World Neurosurg..

[22] Benzel E.C., Mirfarkhraee M., Hadden T., Fowler M. (1987). Holocord Astrocytoma: A Two-Staged Operative Approach. Spine.

[23] Dolen D., Gulsever C.I., Erguven M., Unverengil G., Sabanci P.A. (2025). Holocord Intramedullary Pilocytic Astrocytoma Mimicking Holocord Spinal Abscess: A Case Report and Literature Review. Childs Nerv. Syst..

[24] Epstein F., Epstein N. (1981). Surgical Management of Holocord Intramedullary Spinal Cord Astrocytomas in Children: Report of Three Cases. J. Neurosurg..

[25] Goyal S., Puri T., Julka P.K. (2015). Holocord Low Grade Astrocytoma—Role of Radical Irradiation and Chemotherapy. J. Egypt. Natl. Cancer Inst..

[26] Irikura T., Johki T., Tanaka H., Nakajima M., Yasue M., Sakai H., Nakamura N. (1990). Holocord Astrocytoma—Case Report. Neurol. Med. Chir..

[27] Kiani Salmi S., Dehghanian A., Taherifard A., Dehghan A. (2024). Holocord Pilocytic Astrocytoma in a Young Woman with Intracranial Extension: Case Report and Review of the MRI Characteristics. Spinal Cord. Ser. Cases.

[28] Komotar R.J., Carson B.S., Rao C., Chaffee S., Goldthwaite P.T., Tihan T. (2005). Pilomyxoid Astrocytoma of the Spinal Cord: Report of Three Cases. Neurosurgery.

[29] Lau B.H., Lin M.I., Sung T.C., Wei C.P., Peng H.L., Lee C.C. (1998). Holocord Intramedullary Spinal Cord Astrocytoma: Report of One Case. Zhonghua Min. Guo Xiao Er Ke Yi Xue Hui Za Zhi.

[30] Minehan K.J., Shaw E.G., Scheithauer B.W., Davis D.L., Onofrio B.M. (1995). Spinal Cord Astrocytoma: Pathological and Treatment Considerations. J. Neurosurg..

[31] Nikaido Y., Ohnishi H., Hiramatsu K. (1984). Intramedullary holocord tumor. Report of an autopsy case and review of literature. No Shinkei Geka.

[32] Schittenhelm J., Ebner F.H., Tatagiba M., Wolff M., Nägele T., Meyermann R., Mittelbronn M. (2009). Holocord Pilocytic Astrocytoma--Case Report and Review of the Literature. Clin. Neurol. Neurosurg..

[33] Shafrir Y., Kaufman B.A. (1992). Quadriplegia after Chiropractic Manipulation in an Infant with Congenital Torticollis Caused by a Spinal Cord Astrocytoma. J. Pediatr..

[34] Tedeschi G., Spaziante R., Corriero G., Gambardella A., Pettinato G. (1982). A Case of Pan-Medullary Astrocytoma in a Child. Neurochirurgia.

[35] Vles J.S., Grubben C.P., van Ooy A., Weil E.H. (1990). Holocord Astrocytomas in Childhood. Clin. Neurol. Neurosurg..

